# Cerebral Invasive Aspergillosis in a Case of Chronic Lymphocytic Leukemia with Bruton Tyrosine Kinase Inhibitor

**DOI:** 10.3390/curroncol28010081

**Published:** 2021-02-08

**Authors:** Omar Alkharabsheh, Alhareth Alsayed, Diana M. Morlote, Amitkumar Mehta

**Affiliations:** 1 Mitchell Cancer Institute, University of South Alabama, Mobile, AL 36604, USA; aalsayed@health.southalabama.edu; 2 Department of Pathology, University of Alabama at Birmingham, Birmingham, AL 35233, USA; dmorlote@uabmc.edu; 3 O’Neal Comprehensive Cancer Center at UAB, University of Alabama at Birmingham, Birmingham, AL 35233, USA; amitkumarmehta@uabmc.edu

**Keywords:** CLL, acalabrutinib, invasive aspergillosis

## Abstract

Bruton tyrosine kinase (BTK) inhibitors have become an important therapy for untreated and previously treated patients with chronic lymphocytic leukemia (CLL). Despite improved outcomes, rare adverse events, such as invasive fungal infections, have been reported with the use of first-generation BTK inhibitors. Invasive fungal infections carry a high morbidity and mortality risk. There have been several case reports describing the association between aspergillosis and ibrutinib treatment, but none with acalabrutinib, to our knowledge. In this case report, we describe a patient with CLL who developed an intracranial *Aspergillus fumigatus* infection while receiving acalabrutinib.

## 1. Introduction

Chronic lymphocytic leukemia/small lymphocytic lymphoma (CLL/SLL) is a form of mature B-cell lymphoproliferative neoplasm. The current treatment approach has shifted from chemoimmunotherapy (CIT) to novel agents that have changed patients’ outcomes and survival [1]. Infections, irrespective of the pathogen, can occur due to a dysregulated immune system from CLL or from therapeutic interventions [2].

The advancement of CLL treatment with novel agents such as bruton tyrosine kinase (BTK) inhibitors has resulted in a different profile of adverse events. Although data suggest better tolerability of BTK inhibitors as compared to CIT, infections are still reported with the use of BTK inhibitors. There are several case reports of invasive fungal infections linked to the use of BTK inhibitors [3,4,5,6]. For example, a series of 33 cases of invasive fungal infections in patients receiving ibrutinib alone or in combination have been reported. Aspergillosis was overrepresented in 27 out of the 33 patients and was associated with cerebral localization in 40% of the cases [6]. Another report analyzed 378 patients with CLL and mantle cell lymphoma who received ibrutinib; 84% of the patients received ibrutinib as monotherapy. Invasive fungal infections developed in 16 patients, and eight cases had invasive aspergillosis. Of the eight cases, two involved the central nervous system [7]. A larger observational study by Ruchlemer el al. identified 35 patients with invasive fungal disease while on ibrutinib. Aspergillosis species were identified in 22 patients, and cranial involvement occurred in 60% of the cases [8].

Acalabrutinib is a second-generation BTK inhibitor that is approved for the treatment of CLL. It was designed to exhibit less off-target kinase activity; however, the clinical trials that led to its approval indicated a similar, albeit lower, incidence of adverse events to first-generation BTK inhibitors, such as atrial fibrillation, the risk of bleeding, and infections [9].

Invasive fungal infections are not uncommon in immunocompromised patients. The inhalation of conidia is the most common route of sinopulmonary infections, and central nervous system (CNS) infections can occur after disseminated disease or local extension from an advanced fungal sinusitis [10]. Here, we present a rare case of CNS aspergillus infection secondary to the use of acalabrutinib.

## 2. Case Presentation

A 62-year-old male initially presented with cervical lymphadenopathy, the constitutional symptoms of fatigue and weight loss, and abnormal laboratory values indicating anemia and thrombocytopenia. The baseline white blood cell count was 6.36 × 10^3^/mcL, and the absolute lymphocytic count, 5.71 × 10^3^/mcL. Computed tomography (CT) scans of the chest, abdomen, and pelvis showed enlarged lymph nodes above and below the diaphragm with splenomegaly. An excisional lymph node biopsy from the neck revealed a B-cell lymphoproliferative neoplasm with B-cells expressing CD5, CD19, CD20, CD23, and CD38, consistent with CLL/SLL. Additionally, a bone marrow biopsy and aspiration were performed, indicating the diffuse involvement of CLL/SLL. He had Rai stage IV disease with normal fluorescence in situ hybridization (FISH), including negative t (11;14). Based on the presented symptoms and cytopenia, the decision was made to start CLL/SLL therapy.

He was initiated on an acalabrutinib and obinutuzumab combination as a first-line treatment. He tolerated the first three months of treatment without major complications; no glucocorticoids were used except as a premedication prior to obinutuzumab.

**Figure 1:**
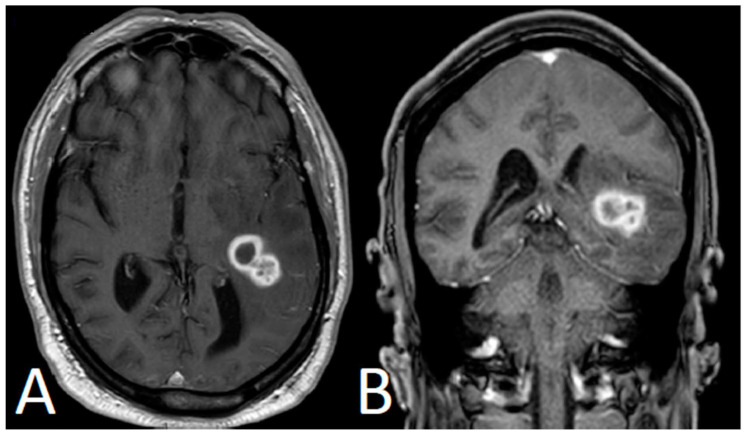
Brain MRI showing lobulated left temporal mass with peripheral enhancement with surrounding cerebral edema (**A**: axial view; **B**: coronal view).

After Cycle 3, the patient’s caregiver noticed episodes of confusion and slurred speech. Brain magnetic resonance imaging (MRI) revealed a left temporal lobe mass with peripheral enhancement measuring 2.4 × 1.7 × 1.7 cm (Figure 1). He did not exhibit symptoms of infection; however, he was admitted to the hospital and was started on broad-spectrum antibiotics. He, then, underwent MRI spectroscopy, which raised the possibility of a neoplastic process (primary or metastasis). A complete re-staging CT scan of the body showed an improvement of the CLL/SLL lymphadenopathy, with no radiologic evidence of a second primary malignancy.

**Figure 2:**
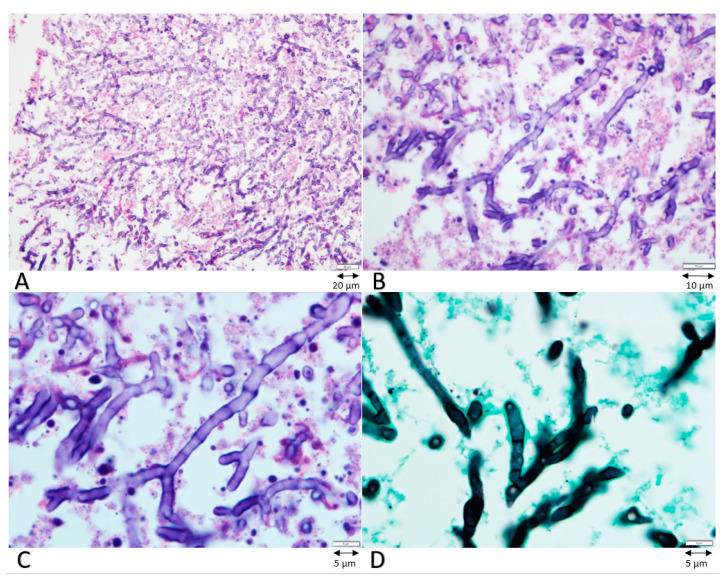
Biopsy images with hematoxylin and eosin stain at different resolutions (**A**–**C**) and silver stain (**D**). Images show septate hyaline hyphae with narrow angle branching compatible with aspergillosis.

He then underwent an emergent stereotactic brain biopsy, which showed gliosis with marked microglial activation and macrophage infiltration. The biopsy was obtained using multiple locations but did not support a specific diagnosis. In the meantime, acalabrutinib and obinutuzumab were withheld; no signs or symptoms of infection other than the CNS symptoms at the time of initial presentation were noted. Due to the possibility of leukoencephalopathy, obinutuzumab was discontinued completely and acalabrutinib was restarted. However, the repeat brain MRI after 3 weeks showed progression of the left temporal lobe lesion, which mandated a second biopsy; the second biopsy was consistent with *A. fumigatus* (Figure 2). Subsequently, the patient was started on amphotericin B and isavuconazonium and showed clinical improvement.

## 3. Discussion

The number of case reports of opportunistic fungal infections associated with BTK inhibitor use is increasing [3,4,5,6]. Those reports are indicating unusual locations of invasive fungal infections such as extrapulmonary *Pneumocystis jirovecii*, disseminated *Cryptococcus neoformans*, and CNS *Aspergillus* [11]. These types of infections are common in patients with severe immunosuppression after high-dose chemotherapy for acute leukemia, or who are undergoing bone marrow transplantation. Little is known about the risk of invasive fungal infection in CLL patients treated with novel agents; we think that the risk of fungal infections is lower in nonconventional therapy or targeted therapy. Nevertheless, some reports indicate an up to 11% risk of fungal infection during frontline ibrutinib therapy or with subsequent relapses [11]. Regarding second-generation BTK inhibitors such as acalabrutinib, zanubrutinib, or Loxo-305 (a covalent BTK inhibitor), which is currently in a clinical trial, only one case report for acalabrutinib has been published with disseminated *Cryptococcus neoformans* including the cerebrospinal fluid [12], and none has been reported for zanubrutinib and Loxo-305.

Infections have a major impact on the course of disease in terms of treatment interruption and worsening quality of life. Infections can result from immune dysregulation leading to lower production of immunoglobulins from plasma cells, less effective cell-mediated immunity, and lymphopenic agents used to treat CLL, such as anti-CD20 monoclonal antibodies, purine analogues, and others [13].

A clear immunological explanation for invasive fungal infections while on ibrutinib is yet to be identified. A preclinical model demonstrated the importance of the BTK pathway in neutrophilic maturation; BTK knockout mice had higher mortality, probably due to the impartment of TLR9–BTK–calcineurin–NFAT pathway activation after phagocytosis [14]. The inhibition of BTK-dependent pathways in CLL leads to macrophage dysfunction and impaired FcyR-mediated phagocytosis [15]. Further evidence that BTK inhibitors have an impact on myeloid cells is a study that hypothesized that myeloid stem cells would express BTK; the study demonstrated that ibrutinib can impair the generation of myeloid precursors [16]. Another possible explanation is the impact of BTK inhibitors on lowering platelet function and degranulation, leading to lower aspergillus virulence [17]. On the other hand, we have observed a partial reconstitution of humeral immunity by ibrutinib that led to improved IgA levels [18].

Comparable adverse event profiles are observed between first- and second-generation BTK inhibitors, such as in the risk of bleeding, atrial fibrillation, hypertension, and skin toxicity. Although the second-generation BTK inhibitors are thought to have less off-target kinase activity, we advise waiting for the results of the head-to-head trials in order to compare safety and tolerability, rather than comparing safety data across trials.

To our knowledge, this is the first case report of an invasive *Aspergillus* infection associated with acalabrutinib. We believe that the risk of invasive fungal infection results from a class effect rather than ibrutinib alone. One essential aspect of *Aspergillus* treatment is the interaction between BTK inhibitors and azoles through CYP3A4 inhibition [19]. This will mandate either dose reduction or the interruption of CLL therapy, which will convey a higher risk of early CLL relapse if BTK is stopped in an incomplete remission state. One limitation to our report is the initial combination with obinutuzumab, as this agent contributes to more lymphodepletion and the suppression of humeral and cellular immunity [20]. The key learning points from this case are (1) to maintain a high index of suspicion when treating CLL patients with BTK inhibitors as single agents or in combination with anti-CD20 monoclonal antibodies, and (2) to request mycological studies when a tissue biopsy is performed, especially if the site of infection carries a high risk of complications, as the brain does.
